# Polyvinylidene Difluoride (PVDF) Hollow Fiber Membrane Incorporated with Antibacterial and Anti-Fouling by Zinc Oxide for Water and Wastewater Treatment

**DOI:** 10.3390/membranes12020110

**Published:** 2022-01-19

**Authors:** Roziana Kamaludin, Lubna Abdul Majid, Mohd Hafiz Dzarfan Othman, Sumarni Mansur, Siti Hamimah Sheikh Abdul Kadir, Keng Yinn Wong, Watsa Khongnakorn, Mohd Hafiz Puteh

**Affiliations:** 1 Advanced Membrane Technology Research Centre (AMTEC), Universiti Teknologi Malaysia, Skudai 81310, Johor, Malaysia; roziana.kamaludin7@gmail.com (R.K.); sumarnimansur90@gmail.com (S.M.); mhafizputeh@utm.my (M.H.P.); 2 School of Chemical and Energy Engineering (SCEE), Faculty of Engineering, Universiti Teknologi Malaysia, Skudai 81310, Johor, Malaysia; lubnaabmajid@gmail.com; 3 Institute of Pathology, Laboratory and Forensics (I-PPerForM), Faculty of Medicine, Universiti Teknologi MARA (UiTM), Cawangan Selangor, Sungai Buloh 47000, Selangor, Malaysia; sitih587@uitm.edu.my; 4 School of Mechanical Engineering, Faculty of Engineering, Universiti Teknologi Malaysia, Skudai 81310, Johor, Malaysia; kengyinnwong@utm.my; 5 Faculty of Engineering, Prince of Songkla University, Hatyai 90110, Songkhla, Thailand; watsa.k@psu.ac.th; 6 School of Civil Engineering, Faculty of Engineering, Universiti Teknologi Malaysia, Skudai 81310, Johor, Malaysia

**Keywords:** polyvinylidene difluoride, zinc oxide nanoparticles, antibacterial, biofouling, anti-fouling membrane

## Abstract

The addition of antibacterial material to hollow fiber membranes improves the membrane anti-biofouling characteristics. Antibacterial membranes were fabricated in this study to improve membrane function while also extending membrane lifetime. Neat polyvinylidene difluoride (PVDF) and PVDF hollow fiber membrane with the incorporation of antibacterial agent zinc oxide (ZnO) nanoparticles with various loading (2.5–7.5 wt.%) were fabricated by using dry/wet spinning method. The membrane structure, particle distribution, functional group, hydrophilicity, and pore size of each membrane were all assessed. The result shows that all ZnO/PVDF hollow fiber membranes have the asymmetric structure with even dispersion of ZnO nanoparticles throughout the membranes. The results showed that increased ZnO loadings considerably improved membrane hydrophilicity, and average pore size, in addition to good performance of pure water flux. Antibacterial testing shows that ZnO incorporated in the membrane matrix and membrane surfaces prevents bacteria that cause biofouling from adhering to the membrane. ZnO/PVDF membrane recorded excellent bovine serum albumin (BSA) rejection at 93.4% ± 0.4 with flux recovery rate at 70.9% ± 2.1. These results suggest that antibacterial ZnO/PVDF hollow fiber membranes are promising in relation to reducing biofouling for various water and wastewater treatment.

## 1. Introduction

Hollow fiber membranes have played a significant part in the development of the membrane field in various industrial sectors due to their advantages such as mechanically self-supporting and easily assembled in modules for various membrane applications [1]. The fact that they provide a wide membrane surface per module volume is the most important feature, in addition to more efficiency in filtration than flat sheet membranes, as hollow fiber membrane can be used in two ways: “inside-out” or “outside-in” [2]. Hollow fiber can be used in a variety of filtration processes, including microfiltration (MF) and reverse osmosis (RO). Membrane bioreactors (MBRs), RO pre-treatment, industrial water/wastewater, juice processing, and biotech applications are all common uses for hollow fiber membranes. When compared to alternative arrangements, hollow fiber membranes have moderate capital costs but high running costs [3]. Furthermore, the main issues with hollow fiber filtration are irreversible fouling and fiber breaking.

Biofouling is the build-up of biological components on membrane surfaces, which reduces the membrane’s permeability. Membrane biofouling is caused by bacteria adhering to membrane surfaces and forming biofilms. Membrane fouling reduced system productivity while increase the energy required for sludge recirculation or gas scouring. As a result, the membrane must be cleaned on a frequent basis to avoid a decrease in membrane durability and, as a result, a higher cost of replacement. Many studies have studied biofouling removal using physical and chemical approaches, but the processes result in increased operating costs and a shorter membrane life span [4]. One of the effective techniques for preventing bacterial attachment is to modify the hydrophobic surface of the membrane. Despite this, hydrophilic polymer membranes are preferred for industrial applications due to their permeability and mechanical strength. As a result, hydrophilic membranes should be developed in order to maximize fouling resistance while maintaining membrane performance. Numerous studies have been conducted to incorporate a number of materials onto the membrane surface, such as nanoparticles, antimicrobial polymers, and antibiotics [5,6].

The application of antibacterial nanoparticles in hollow fiber membranes is an innovative membrane technology. There are a large number of antibacterial agent such as zinc oxide (ZnO), titanium oxide (TiO_2_) silver (Ag), graphene oxide (GO), and copper (Cu) that have been used in membrane anti-biofouling [7]. The presence of antibacterial agent combats the microorganism attachment, hence, preventing the build-up of biofoulant on surface generally and membrane pores specifically, thus subsequently reducing the membrane biofouling [8]. According to literature, the improvement in membrane antibiofouling is related to a reaction between metal oxides and microorganisms on the membrane surface, which prevents biofilm development [9]. The presence of metal oxide on the membrane surface is intended to prevent the formation of biofilm from initial contact and production stage. Apart from the superior antibacterial features that eventually improved the anti-biofouling properties of the membrane, the presence of antibacterial agent improved the membrane hydrophilicity and its permeability. In addition, the presence of nanoparticles on the membrane surface and membrane structure can contribute to increased mechanical strength as well as improved performance as a result of modifications to the surface and internal pore structure.

Recently, ZnO nanoparticles have come to the forefront as one of the most promising materials to resist bacterial infections. ZnO is a familiar anti biofouling agent used to mitigate the membrane biofouling in water treatment process [10]. ZnO has a broad spectrum of antibacterial activities including on *Bacillus subtilis*, *Staphylococcus aureus*, *Staphylococcus epidermidis*, *Streptococcus pyogenes*, and *Enterococcus faecalis* [11,12]. Previously, an inhibitory effect of ZnO on *Escherichia coli* and *Listeria monocytogenes* has been reported as well [13]. In nanoscale, ZnO possesses antibacterial qualities, making it a suitable candidate for a variety of applications. Furthermore, it is robust under rigorous manufacturing conditions and is considered a safe substance for humans and animals [14]. Previously, Hong and He [15] used a blending approach to create PVDF–ZnO composite membranes. The results revealed that adding ZnO nanoparticles reduced water contact angles, resulting in improved antifouling properties. Meanwhile, Zhao et al. [16] developed PES–ZnO composite membranes and discovered that, as compared to pristine PES membranes, PES–ZnO composite membranes had a more porous membrane structure and high heat stability. Furthermore, Kim et al. [17] discovered considerable photocatalytic/antimicrobial activity in the creation of the polyurethane (PU)–ZnO composite material, which was suitable for a prospective application in organic pollutant degradation and wastewater purification.

Previously, incorporation of ZnO onto polymeric membrane was mostly undertaken on flat-sheet configuration with low loadings of ZnO nanoparticles. Therefore, the goal of this research is to fabricate an effective anti-bacterial hollow fiber membrane (AHFM) with high loadings of ZnO nanoparticles to prevent membrane biofouling for water and wastewater applications. In this regard, hollow fiber membranes feature a very high packing density because of the small strand diameter. Because of the flexibility of the strands, certain filter configurations are possible that cannot be achieved in other filtration configurations. Interestingly, the presence of antibacterial agent ZnO will enhance the membrane performance by inhibiting the growth and reproduction of the microorganism and the development of bio-film. As previously mentioned, ZnO has been widely employed as an antibacterial agent because of its high antibacterial activity and broad antibacterial spectrum [10,11,12,13]. The anti-biofouling properties of the fabricated AHFM would avoid an enormous decrease in membrane permeability, thereby reducing the chemical or physical cleaning of membrane modules. This situation prolongs the membrane usage and eventually contributes to cost saving both of the operation and maintenance in the AHFM application.

In this current study, ZnO/PVDF AHFM with various ZnO loadings has been developed employing a fixed spinning condition via dry/wet phase inversion technique. The membrane was characterized for its morphology by scanning electron microscopy (SEM), particle distribution by energy dispersion X-ray analysis (EDX) as well as surface roughness by atomic force microscopy (AFM), in addition to the hydrophilicity, functional group, and pore size analysis. The antibacterial properties of the ZnO/PVDF AHFM were investigated via agar dilution method followed by performance evaluation of the membrane. Preparing anti-biofouling ZnO/PVDF has several advantages including key method for dealing with widespread biofouling caused by a range of biofoulants. The outcome of this study is to provide the potential of ZnO/PVDF as antibacterial hollow fiber to reduce membrane biofouling, thus increasing the membrane lifetime for various water and wastewater treatment.

## 2. Materials and Methods

### 2.1. Membrane Material

Polyvinylidene difluoride (Solvay, Rue de Clichy, Paris, France), zinc oxide (Sigma Aldrich, MO, USA), *N*,*N*-Dimethylacetamide (QReC Chemicals, New Zealand) were used as polymer based, antibacterial agent and solvent polymer, respectively. Prior to preparation of dope all materials were dried for 24 h at 50 °C for moisture removal.

### 2.2. Fabrication of ZnO/PVDF Antibacterial Hollow Fiber Membrane (AHFM)

The neat PVDF dope solution and ZnO/PVDF dope solutions with different ZnO loadings were prepared according to Table 1.

The required amount ZnO and DMAc were stirred in a Scott bottle until homogenous, followed by the addition of PVDF at the required amount. The polymer dope solutions were then cooled down to room temperature, and were degassed overnight in a room temperature ultrasonic bath system before spinning.

The fabrication of neat PVDF and ZnO/PVDF hollow fiber membrane at various ZnO loadings employed a fixed spinning parameter via dry/wet spinning technique. All dope solutions were pumped into a stainless steel reservoir followed by extrusion into a spinneret to form hollow fiber membranes. The dope solution flowrate was 8 mL/min. Distilled water was used as the bore fluid with a flow rate of 8 mL/min. The hollow fiber membrane was drawn into a coagulation bath after passing through an air gap of 10 cm at room temperature with drum take up speed was set at 3 rpm [18,19]. To preserve the pore structure, the as-spun fibers were immersed in tap water overnight to complete the solvent-nonsolvent exchange process. The as-spun fiber was then soaked in 50% ethanol and 100 percent ethanol for 1 h, respectively. The membranes were then air-dried for 72 h and kept in a clean and dry place until further used.

### 2.3. Characterization Study of ZnO/PVDF Antibacterial Hollow Fiber Membrane (AHFM)

#### 2.3.1. Morphological Analysis

The morphology of MNeat and all ZnO/PVDF AHFM prepared was explored by scanning electron microscopy (SEM; Model: TM 3000, Hitachi, Tokyo, Japan). Energy-dispersion X-ray (EDX) analysis was undertaken to investigate the ZnO distribution on the membrane surface and membrane structure, meanwhile Atomic Force Microscopy (AFM; Model: SE-100, Park System) was used to study the surface roughness and membrane topography.

#### 2.3.2. Contact Angle

Contact angle goniometer (OCA 153C, Dataphysic) was used to evaluate the degree of hydrophilicity of all fabricated membrane. An amount of 100 µL of contact liquid (deionized water) is sessile dropped on the surface of each sample. The measurements of contact angle were obtained at various locations on the surface of sample.

#### 2.3.3. Porosity and Pore Size

The intrusion of mercury into the membrane structure under required pressure via automated mercury porosimeter (Model: AutoPoreTM IV Series, Micromeritics) was used to determine the porosity and pore diameter of all samples. For the MIP analysis, the sample was cut into pieces with a total weight of not less than 0.25 g.

#### 2.3.4. Fourier Transform Infra-Red (FTIR) Spectroscopy

Perkin Elmer FTIR attenuated total reflection (ATR) spectrophotometer and diamond ATR sampling accessory was used to investigate the FTIR spectra of all samples at wave numbers ranging from 650 to 4000 cm^−1^.

### 2.4. Antibacterial Properties Testing ZnO/PVDFAHFM

The antibacterial test was performed according to the agar dilution method. The bacteria were grown on the agar plate at 30 °C for 24 h. ZnO/PVDF AHFM were then mounted on the agar plate followed by the incubation of the agar plate. Generally, the antimicrobial element diffuses into the agar and prevents the test bacterium from germinating and growing. The inhibition zone of the bacteria was measured from the center of the disk to the edge of area with zero growth.

### 2.5. Membrane Performance: Pure Water Flux, BSA Rejection and Antifouling of ZnO/PVDF Antibacterial Hollow Fiber Membrane (AHFM)

Initially, membranes were compacted with pure water (distilled water/RO) at 0.35 MPa for 30 min to get a constant flux. Pure water flux measurement was tested up to 0.3 MPa and the flux was measured according to Equations (1) and (2) below:

(1) $$F=\frac{V}{\mathrm{Axt}}$$

(2) A=πdoL

where:

F = membrane flux (L/m^2^ h)
V = volume of permeate at time (t)
A = effective filtration area of the membrane (m^2^),
do = outer diameter of hollow fibers (cm)
L = effective length of hollow fibers (cm).

Bovine serum albumin (BSA: Mw: 66 KDa) at 500 ppm was used as the foulant solution. The different of BSA content in feed and permeate solution was measured using UV-Visible spectrophotometer (DR5000, HACH) at 280 nm. All filtration was performed at pressure 0.3 Mpa. Membranes rejection against BSA (*R*%) was determine using the Equation (3) below:

(3) $$R\%=\left(\frac{C_{0}-C_{1}}{C_{0}}\right)\times 100$$

where

C_0_ = initial concentration of feed solution
C_1_ = concentration value of the permeate solution.

The fouling recovery of Mneat and all AHFM will be investigated by three-step filtration method with BSA as a model foulant. During the experiment, the pure water flux was performed for 1 h and then replaced with 500 ppm of BSA solution for another hour. The fouled membranes were washed with pure water for 30 min prior to evaluation of pure water flux again. The Flux recovery ratio (FRR) value (Equation (4)) of all AHFM was compared with MNeat membrane.

(4) $$\mathrm{FRR}\left(\%\right)=\frac{Jw2}{Jw1}\times 100$$

where

*Jw*1 = pure water flux
*Jw*2 = pure water flux of the cleaned membrane.

## 3. Results

### 3.1. Physical Properties

**Figure 1:**
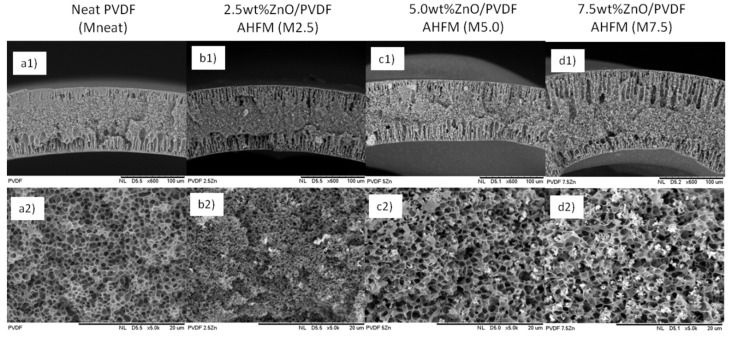
Cross-sectional images of (**a1**,**a2**) neat PVDF(MNeat) (**b1**,**b2**) 2.5 wt.% ZnO/PVDF (M2.5) (**c1**,**c2**) 5.0 wt.% ZnO/PVDF (M5.0) and (**d1**,**d2**) 7.5 wt.% ZnO/PVDF (M7.5) AHFM at different magnification.

The physical appearance of all samples was analyzed in terms of surface and cross-section structure at different magnification levels of SEM analysis. The result shown in Figure 1 depicts the membranes with different concentration which PVDF/DMAc concentration of (21/79 wt.%) designated as MNeat and ZnO/PVDF/DMAc concentration of (2.5/21/76.5 wt.%), (5/21/74 wt.%) and (7.5/21/71.5 wt.%), which was designated as M2.5, M5.0 and M7.5, respectively. The distinctive sandwich-like shape of all developed membranes can be seen in cross-sectional morphological images, which comprise of the finger-like structure developed at the inner and outer membrane layers, and the sponge-like structure developed in the middle membrane layer (Figure 1a–d). Similarly, Shi, Liu and Xue [20] discovered the identical dimension and main structure of antibacterial PVDF hollow fiber membrane by doping Ag-loaded zeolites. All membranes possessed finger-like structures ran through the inner and outer walls of the hollow fiber membranes with sponge-like structure between the little voids and the finger-like structures. The formation of such structure was due to quick precipitation at both the inner and outer walls, which resulted in a long finger-like structure, and delayed precipitation, which resulted in a sponge-like structure.

Interestingly, in this study, the formation of the finger-like, is more regular with longer size in all AHFM membranes than the pristine PVDF membrane. The membrane loaded with higher ZnO loadings yield more open macrovoids structure compared to MNeat hollow fiber. As shown in Figure 1, M2.5 possesses better shape with long finger-like structures in the membrane structure, as compared to MNeat, M5.0 and M7.5 membranes. In this regard, the addition of ZnO loadings would definitely increase the hydrophilicity of the outer membrane, hence, attracting large amounts of the water flow and enlarging the volume of the nucleus. In contrast, high loadings of ZnO cause too much water flow to the nucleus, hence, creating more macrovoids. Previously [21], high loadings of nanoparticles attribute to the increased number and size of macrovoids of the nanocomposite membrane. The results revealed that increasing the amount of nanocomposite to 0.75 wt.% increased the mean void size substantially. The drastic alteration in the morphology of the nanocomposite membranes when compared to a pure PVDF membrane is believed due to the blending of modified ZnO nanoparticles into the casting solution. In the meantime, Figure 1(a2–d2) shows the middle region of membrane structure. The figure shows that the resultant membranes also have porous sponge-like structures, accordingly with increasing loadings of ZnO nanoparticles. Increased pore size accordingly with the increase in the amount of ZnO loadings, was due to the attraction of large water flow into the membrane pores resulting from the increasing in the membrane hydrophilicity [22]. Long finger-like and porous membrane structure may increase the water flux through the membrane.

**Figure 2:**
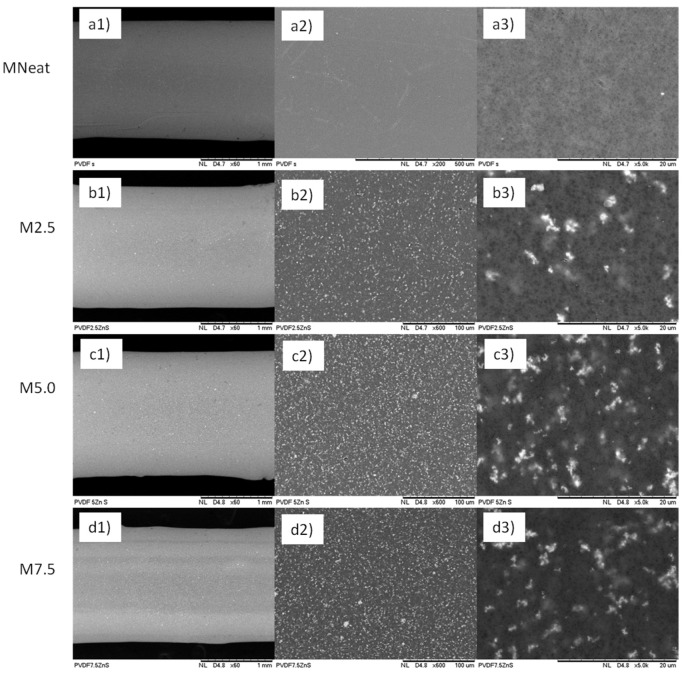
Surface morphologies of (**a1**–**a3**) MNeat (**b1**–**b3**) M2.5 (**c1**–**c3**) M5.0 and (**d1**–**d3**) M7.5 hollow fiber membranes at different magnification.

The outer-surfaces image of the neat PVDF and ZnO/PVDF AHFM with different loadings of ZnO are shown in the Figure 2. The surface of all membranes is composed of porous structure with uniform pore distribution of ZnO nanoparticles across the membranes surface (Figure 2(b2–d2)). In addition, as shown in the Figure 2((b3–d3)), the amount of ZnO particles distributed on the membrane surface was increased with increasing ZnO loadings in the polymer dope suspension. However, greater surface tension between the solvent (DMAc) and high ZnO loadings produce agglomeration of the ZnO on the membrane surface [23]. When DMAc comes into contact with the hydrophilic ZnO, it causes colloidal instability, which increases ZnO agglomeration throughout the fabrication process [18,24]. This result is in line with previously published studies where the agglomeration of nanoparticles was observed on the membrane surface fabricated with high loadings of nanoparticles [19,21].

The diameter of all membranes was measured during the SEM analysis at magnification X60, and the thickness was calculated from the OD and ID obtained. The summary of OD, ID and membrane thickness was listed in the Table 2. The thickness of all fabricated ZnO/PVDF AHFM ranged from 290–380 μm. The incorporation of ZnO into the membrane surface increased the membrane thickness due to the increased viscosity of the polymer dope solution, which reduced the flow of the dope solution during the fabrication process resulting to thicker membrane layer.

**Figure 3:**
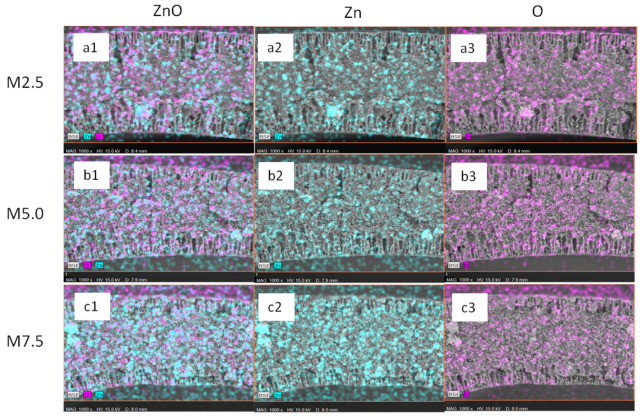
EDX mapping of (**a1**–**a3**) M2.5 (**b1**–**b3**) M5.0 and (**c1**–**c3**) M7.5 AHFM.

The presence of ZnO on the membrane surface within the matrix structure of all ZnO/PVDF AHFM was verified by EDX imaging (Figure 3). From the spectrum analysis, it can be seen that the Zn element and O element increased with the increasing nanoparticle loading. Furthermore, ZnO dispersed evenly on the surface as well as in the membrane matrix, even though there is some agglomeration of the ZnO NPs at high loadings configuration. The even distribution of ZnO NPs will be a great advantage in preventing the build-up of microorganism during the treatment process, which subsequently reduce the membrane fouling events.

Table 3 summarizes the contact angle value, average pore size and porosity of all fabricated membranes. The contact angle value of all membranes was in a range of 70.93°–78.06°. From the table, the value of contact angle decreased as the ZnO loading concentration increased. Membrane M7.5 possesses the lowest CA value as compared to others due to the highest composition of ZnO nanoparticles. In this regard, the hydrophilicity of a membrane increases as the distribution of hydrophilic properties on the membrane increases [25]. Bojarska et al. [26] observed a reduction in contact angle in water from 135◦ to 107° after plasma treatment of polypropylene membranes. The implantation of hydrophilic functional groups (hydroxyl or carboxyl) into the membrane surface causes a reduction in advanced contact angle (increased hydrophilicity) [26,27]. The water flux and antifouling properties of membranes can be affected by their high hydrophilicity. Most of the organic fouling and bacteria are hydrophobic in nature, thus the settlement of the foulants on hydrophilic surface is not very likely to happen. Interestingly, higher ZnO loadings also increase the average pore size. The average pore size of M2.5, M5.0 and M7.5 AHFM ranged from 75.22 to 161.54 nm, respectively. The formation of the larger pore size was due to the presence of ZnO, which decreased the interaction between the polymer (PVDF) and solvent (DMAc), thus increasing the exchange rate of the polymer dope solution and coagulation bath. According to the literature, the viscosity of polymer dope solution was another factor affecting the pore size of the membranes during the fabrication. High dope solution viscosity slowed mass transfer between the solvent and coagulation bath, resulting in increased pore size as evidence by SEM images (Figure 3(a2–d2)). In contrast, the porosity of all AHFM decreased as compared to MNeat, in line with the findings of previous literature wherein they reported that neat membrane possessed the highest porosity value [28]. In addition, they also reported a decrease in membrane porosity of nanocomposite membrane due to an increase in nanoparticles concentration. In addition, decrease in porosity is linked to an increase in viscosity, resulting in the membrane losing most of its finger-like layer and forming a denser sponge-like layer [29]. Furthermore, a decrease in porosity of the AHFM might be strongly related to the agglomeration of ZnO on the membrane surface, as well as in the membrane matrix, as the entrapping of ZnO agglomeration filled the membrane pores, which act as the resistance during the permeation process. A decrease in porosity was also observed in the film with a higher ZnO loading due to the agglomeration and bulky ZnO particles in the casting solution. This could have disrupted the polymer network structure and restricted its motions, resulting in the production of a film with the least porosity [30].

**Figure 4:**
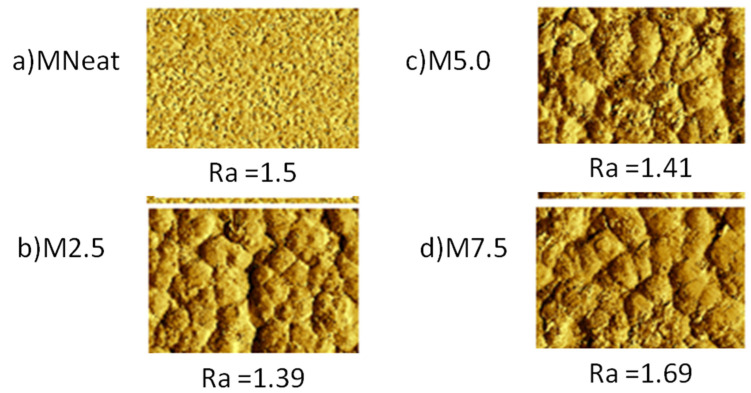
AFM images of (**a**) MNeat (**b**) M2.5 (**c**) M5.0 and (**d**) M7.5 membranes.

AFM analysis on the surface structure of the MNeat, M2.5, M5.0 and M7.5 AHFM was depicted in Figure 4. From the analysis, the Ra values for the newly fabricated M2.5, M5.0 and M7.5 AHFM membranes were 1.39 ka.u., 1.41 ka.u. and 1.69 ka.u. respectively. Meanwhile, the Ra values for MNeat membrane were 1.5 ka.u. The addition of ZnO at 2.5 wt.% and 5.0 wt.%, decreased the surface roughness to 1.39 ka.u and 1.41 ka.u, respectively. Initially, the presence of ZnO in the membrane structure filled the membrane pores and producing a smooth surface [31]. On the other hand, a further increase in ZnO loadings (7.5 wt.%) created a rougher surface than the MNeat, which may be associated with the buildup of excessive ZnO nanoparticles at particular pores and valley, resulting to the rougher surface area [32]. In addition, the agglomeration of the ZnO nanoparticles at higher loading also contributed to the rougher surface formation. With regard to the performance, smoother membrane is more favorable as smoother surfaces would promote antifouling capability due to less foulant build-up on the membrane surface [33]. The higher loading of ZnO induces the high hydrophilicity, high pore size, low porosity and high thickness, which induces the higher permeate flux and less fouling.

### 3.2. Chemical Properties

**Figure 5:**
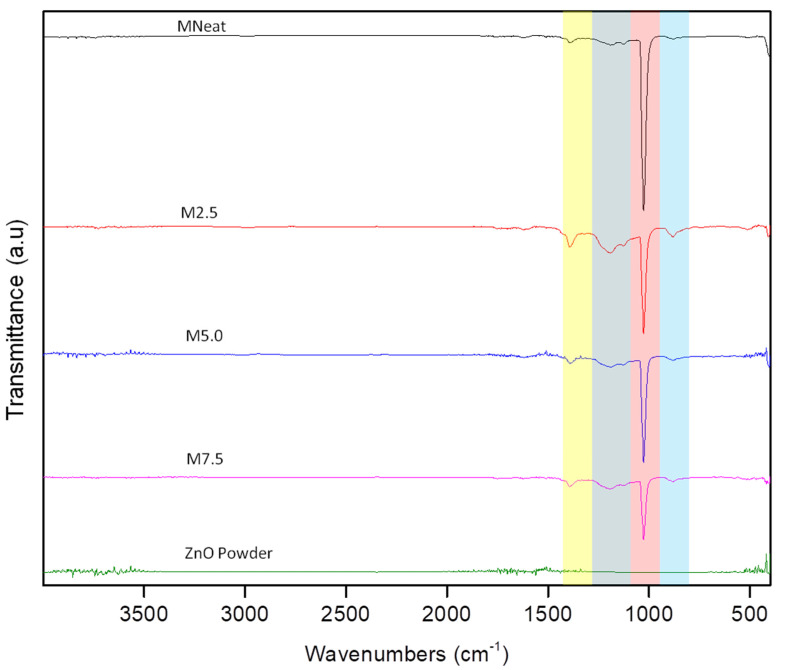
FTIR spectra of MNeat, M.25, M5.0, and M7.5 hollow fiber membranes.

FTIR spectra of MNeat and all AHFM in the range 550–4000 cm^−1^ are shown in Figure 5, below. The stretching vibration of the CF bond was attributed to the strong absorbance at 1191.88 cm^−1^, while the peaks at 1392.69, 1026.42, and 881.01 cm^−1^ were characteristic stretching vibrations of CF2 groups of PVDF polymorphic phase [34]. The 2.5 M has greater peak intensity than the others at 1392.69 cm^−1^. The addition of ZnO may aid in the crystallization of the -PVDF phase. Furthermore, no additional bands appeared after adding ZnO to the PVDF matrix, demonstrating that ZnO solely interacted physically with PVDF using this membrane fabrication process.

### 3.3. Antibacterial Activity of ZnO/PVDF AHFM

**Figure 6:**
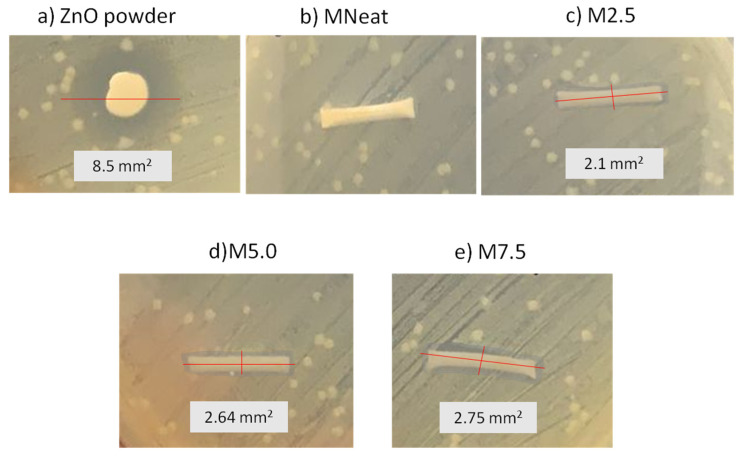
Bacterial growth inhibition of (**a**) ZnO powder (**b**) neat MNeat (**c**) M2.5 (**d**) M5.0 and (**e**) M7.5 AHFM.

The antibacterial testing of all AHFM used gram-negative bacteria Escherichia coli (*E. coli*) as the model biofoulant. Results of the antibacterial test of all tested membranes were depicted in Figure 6. From the figure, the inhibition zone of ZnO powder is 8.5 mm^2^. Antimicrobial activity of ZnO particles has been demonstrated in several studies [35,36]. The antibacterial properties are due to its oxidative activity whereby the inhibition of bacteria growth was linked to the high rate of oxygen generation on the ZnO surface [37]. The bacterial inhibition could be related to direct interaction of ZnO with bacterium cell walls, resulting in the destruction of bacterial cell integrity [12,37]. The antibacterial activity of all ZnO/PVDF AHFM shown in the Figure 6b–d is indicated by the clear zone surrounding the membrane samples. The area measured was 2.1 mm^2^, 2.64 mm^2^ and 2.75 mm^2^, respectively, for M2.5, M5.0, and M7.5 AHFM indicating that the bacterial growth decreased with increased ZnO loadings. In this work, M7.5 inhibited the most germs, implying that the antibacterial properties of PVDF/ZnO were in accordance with ZnO loadings. This result was in line with Kochkodan et al. [4], who showed reduced *E. coli* adherence on the hydrophilic membrane surface. Previously, Bojarska et al. [26] determined the antibacterial properties of polypropylene membranes with ZnO nanowires (PP/ZnO) against model gram (+) and gram (−) bacteria, namely, *B. subtilis* and *E. coli*, respectively. The antibacterial testing was made in comparison with polypropylene membranes (PP). From the results, there is no obvious zone of reduced growth on the polypropylene membrane for either bacteria strain. Meanwhile, a clear zone of inhibited growth can be seen on the PP/ZnO on *B. subtilis*, whereas for *E. coli*, there was essentially no zone of decreased growth. The antibacterial properties, which were further determined in liquid medium, show that the inclusion of ZnO on the membrane surface reduces bacteria cell density after 24 h of *E. coli* incubation regardless of the initial concentration employed. Based on decrease or disappearance of FTIR peak, cell lysis and partial mineralization of cell organic compounds by zinc oxide nanowires can be attributed to the decay of lipids, carbohydrates and amino acids as well as decomposition of secondary to primary amides. Therefore, membranes with ZnO are thought to have good antibacterial/bacteriostatic capabilities against both gram (+) and gram (−) bacteria. This could indicate that the antimicrobial membrane has been successfully developed in this study too.

### 3.4. Flux Performance and Rejection Capability

**Figure 7:**
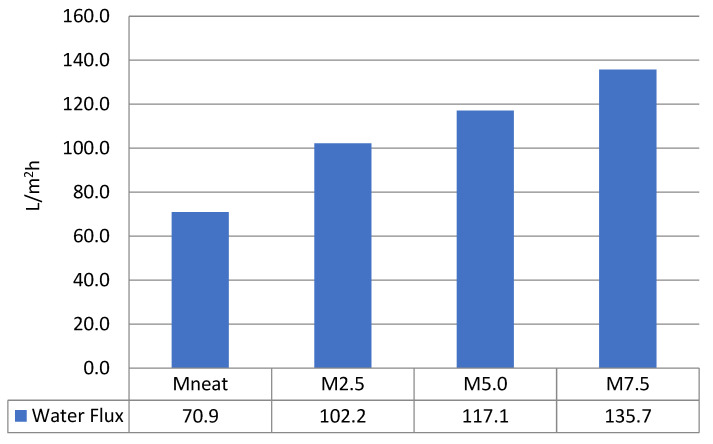
Pure water flux of M2.5, M5.0, and M7.5 AHFM in comparison with MNeat membrane (*n* = 3).

Pure water flux performance was performed for all fabricated ZnO/PVDF AHFM in comparison with neat PVDF membrane. Pure water flux results presented in the Figure 7 was range from 70.9 ± 4.6 L/m^2^ h to 135.7 ± 0.8 L/m^2^ h, recorded at 0.3 MPa. The flux performance is in accordance with the membrane structure. Water flux has a direct relationship with membrane wettability, number of pores on the membrane surface and pore size in general. The presence of ZnO on the membrane surface and membrane structure has increased the water flux from 70.9 ± 4.6 L/m^2^ h to 102.2 ± 1.6 L/m^2^ h by M2.5 as compared to the MNeat. Interestingly, the pure water flux performance increased accordingly with increased loadings of ZnO. The highest water flux recorded was to 135.7 ± 0.8 L/m^2^ h by M7.5. This result indicates that the existence of ZnO significantly increases the wettability as confirmed by increase hydrophilic properties of the membranes (results shown on Table 3) and higher pore size. In addition, the improved water flux in all AHFM was attributed to the influence of even distribution of ZnO, which resulted in a high average pore size, hence better water permeation through the membrane wall. Similar findings were obtained in previous studies, which reported high water flux performance by modified membranes as compared to the neat membrane [38].

**Figure 8:**
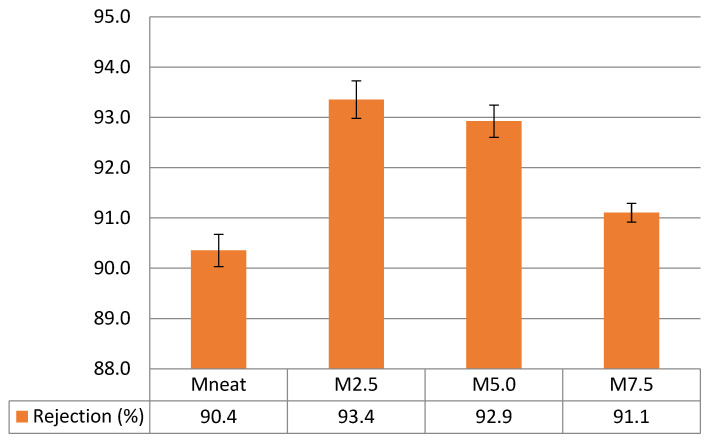
BSA rejection of M2.5, M5.0, and M7.5 AHFM in comparison with MNeat membrane (*n* = 3).

In the meantime, the rejection rate of ZnO/PVDF AHFM in comparison with neat PVDF membrane was examined using a BSA solution (mw: 66 kDa). The rejection rate of MNeat and all AHFM are summarized in Figure 8. There is no significant changes in the rejection rate (*R*%) of all samples. The highest rejection of BSA was by M2.5 with 93.4% ± 0.4. The rejection rate was 3.2% higher than neat PVDF membrane which recorded 90.4% ± 0.3 of BSA rejection. BSA has a stronger affinity for hydrophobic surfaces than hydrophilic surfaces [39]. Therefore, the greater BSA rejection by M2.5 could be attributed to increased hydrophilicity of M2.5, which hinders BSA adsorption. On the other hand, the BSA rejection rate was slightly decreased with increasing of ZnO loadings in the membrane. M5.0 and M7.5 AHFM recorded BSA rejection rate at 92.9% ± 0.3 and 91.1% ± 0.2, respectively. This might br due to the larger average pore size of both membranes reported previously in the Section 3.1. Furthermore, high loadings of ZnO results in the nanoparticles agglomeration, thus increasing the hydrophobicity of the membrane and reducing its performance [40]. Interestingly, the rejection rate of both membranes still exceed 90%, indicating that they fulfill the molecular weight cut-off requirement in filtration to determine pore size distribution and membrane retention capacities [41].

### 3.5. Antifouling Performance

**Figure 9:**
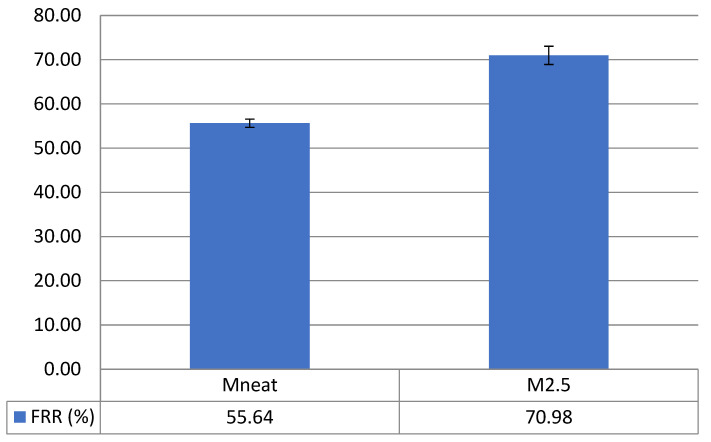
Flux recovery rate of M2.5 AHFM in comparison with MNeat membrane.

The fouling recovery was determined using a three-step filter with pure water flux and BSA as a model foulant to assess antifouling efficacy of the fabricated membranes. In this regard, the antifouling performance was tested on M2.5 AHFM in comparison with MNeat membrane; M2.5 is more favorable because it has smaller pore size compared to M5.0 and M7.5, which gives a better filtration of contaminant as confirmed by excellent BSA rejection. The FRR of both membranes was depicted in Figure 9. The FRR value in the MNeat membrane and M2.5 AHFM was 55.64% ± 0.9 and 70.9% ± 2.1, respectively. Low FRR value of the MNeat was due to its moderate hydrophobicity, which increased the deposition of BSA on the membrane surface. Conversely, increased hydrophilicity of M2.5 AHFM was due to the influence of OH groups in ZnO resulting in high FRR. Furthermore, the antibacterial property of ZnO reduces the adsorption of model foulant on the membrane surface to enhance the membrane permeability. In this regard, the antibacterial properties of ZnO reduce or inhibit the growth of foulants and microorganism, thus preventing the particle build-up, thereby decreasing the fouling of the membrane.

## 4. Conclusions

This research described the preparation of ZnO/PVDF AHFM at different ZnO loadings. The morphology of ZnO/PVDF yield more porous structure compared to the MNeat due to the presence of ZnO nanoparticles. From the findings, it was evident that the membrane was best produced using the 2.5 wt.% of ZnO loading because it yields long finger-like structure with average pore size 75.22 nm, high hydrophilicity, smooth membrane surface and good inhibition zone, which shows the excellent antibacterial activity. Furthermore, M2.5 possesses ultrafiltration configuration with average pore size of 75 nm, which is better for the filtration of viruses, bacteria and suspended solids, in addition to good water flux performance at 102.2 ± 1.6 L/m^2^ h and excellent BSA rejection with 93.4% ± 0.4. Most importantly, M2.5 recorded high FRR value at 70.9% ± 2.1. The antibacterial properties of ZnO minimize the model foulant adsorption on the membrane surface and in the pore to enhance the membrane permeability. In this regard, the presence of antibacterial ZnO nanoparticles on the membrane surface and membrane structure hinders the growth of microorganisms, preventing particle build-up and thereby reducing membrane biofouling. In conclusion, ZnO/PVDF AHFM is a promising solution for inhibiting biofouling for real water and wastewater treatments.

## Figures and Tables

**Table 1:** Compositions of the polymer dope solutions.

Composition (wt.%)	ZnO/PVDF AHFM			PVDF Hollow Fiber Membrane
	M2.5	M5.0	M7.5	MNeat
ZnO	2.5	5	7.5	-
PVDF	21	21	21	21
DMAc	76.5	74	71.5	79

**Table 2:** OD, ID and thickness of the neat PVDF and different concentrations of ZnO/PVDF membranes.

Membranes	Outer Diameter (μm)	Inner Diameter (μm)	Thickness (μm)
MNeat	1750	1490	260
M2.5	1700	1410	290
M5.0	1810	1470	340
M7.5	1780	1400	380

**Table 3:** CA (^o^) value, average pore size and porosity of MNeat and all AHFM.

Membranes	CA (°)	Porosity (%)	Average Pore Size (nm)
MNeat	79.27°	52.60	72.66
M2.5	78.06°	51.38	75.22
M5.0	74.61°	48.90	132.72
M7.5	70.93°	44.06	161.54

## Data Availability

The raw/processed data required to reproduce these findings cannot be shared at this time as the data also forms part of an ongoing study.
